# The Evolving Role of Selenium in the Treatment of Graves' Disease and Ophthalmopathy

**DOI:** 10.1155/2012/736161

**Published:** 2012-01-19

**Authors:** Leonidas H. Duntas

**Affiliations:** Endocrine Unit, Evgenidion Hospital, University of Athens, 20 Papadiamantopoulou Street, 11528 Athens, Greece

## Abstract

Graves' disease (GD) and ophthalmopathy (GO) are organ-specific autoimmune-inflammatory disorders characterized by a complex pathogenesis. The inflammatory process is dominated by an imbalance of the antioxidant-oxidant mechanism, increased production of radical oxygen species (ROS), and cytokines which sustain the autoimmune process and perpetuate the disease. Recently, selenium, which is a powerful antioxidant, has been successfully applied in patients with mild GO, slowing the progression of disease, decreasing the clinical activity score, and appreciably improving the quality of life. The mechanisms of selenium action are variable. The aim of this review is to summarize the actions of selenium in GD and GO. Selenium as selenocysteine is incorporated in selenoproteins, such as glutathione peroxidase which catalyzes the degradation of hydrogen peroxide and lipid hydroperoxide that are increasingly produced in hyperthyroidism. Moreover, selenium decreases the formation of proinflammatory cytokines, while it contributes, in synergy with antithyroid drugs, to stabilization of the autoimmune process in GD and alleviation of GO. It is now to be clarified whether enforced nutritional supplementation has the same results and whether prolonging selenium administration may have an impact on the prevention of disease.

## 1. Introduction

Observed and briefly described, though not published, by Parry in the late 1700s, Graves' disease (GD) was definitively identified and documented by Robert Graves in 1835 and classically described by von Basedow in 1840 [1–3]. GD is an autoimmune disease characterized by the activation of autoantibodies against the TSH receptor (TRAB), leading to excessive thyroid hormone production [4]. GD manifests, interalia, via thyrotoxicosis and extrathyroid involvement often entailing orbitopathy (GO) and, rarely, dermopathy (pretibial myxedema) and acropathy. Moreover, the TRAB, by stimulating cyclic adenosine monophosphate (AMP), cause proliferation and hyperplasia of the thyroid follicular cells resulting in enlargement of the gland, frequently the first sign of the disease, the swelling ranging from slight to marked [5]. Clinically, the thyroid is firm in consistency and tender in patients with a greatly enlarged goiter, while palpation lobulations are also commonly detected which can be mistaken for nodules.

No single gene has been pinpointed as causing GD, a disease which is most prevalent in women between the ages of 20 and 50 years. However, it has been associated with certain MHC Class II HLA alleles depending on the racial group, for example, HLA-DR3 in whites [4]. An association of GD with polymorphisms of the cytotoxic T-lymphocyte antigen 4 (CTLA-4) gene has also been established, suggesting a functional role of CTLA-4 in autoreactive T cells [4, 5].

A combination of genetic and environmental factors is responsible for the initiation of autoimmunity. Interactions between genetic and environmental factors are underscored by the existing associations linking age at diagnosis, goiter, disease severity, smoking, and family history [6]. In addition, iodine repletion in iodine-deficient areas is usually accompanied by an increased incidence of GD due to the Jod-Basedow phenomenon. Stress is also thought to be a significant factor precipitating GD in susceptible individuals [7], while smoking is well established as being linked to GO but not to GD [8].

Treatment modalities of GD consist of administration of antithyroid drugs, radioiodine therapy, or surgery. Radioiodine therapy, is favored only in USA, whereas antithyroid drugs, including methimazole, carbimazole, and propylthiouracil, comprise first choice treatment in the rest of the world. Nevertheless, according to a recent study examining the frequency of antithyroid drug prescription in USA, methimazole (MMI) has lately become the most frequently prescribed antithyroid drug, indicating a clear shift towards pharmacological treatment as the primary treatment option in GD [9]. Treatment should be planned for a period of at least 12 months, and patients are usually becoming euthyroid within this timeframe; nevertheless, the duration of the remission period is unpredictable, since the disease is marked by cycles of remission and relapse of variable duration [4].

Recently, evidence has emerged indicating that selenium administration could be effective and safe in patients with GD and with mild forms of GO [10].

The aim of this paper is to briefly evaluate the current knowledge concerning the pathogenesis of GD and GO and discuss the evolving role of selenium within the context of its potential as a therapeutic means of intervention in these disorders.

## 2. Pathogenesis of GD and GO

**Figure 1:**
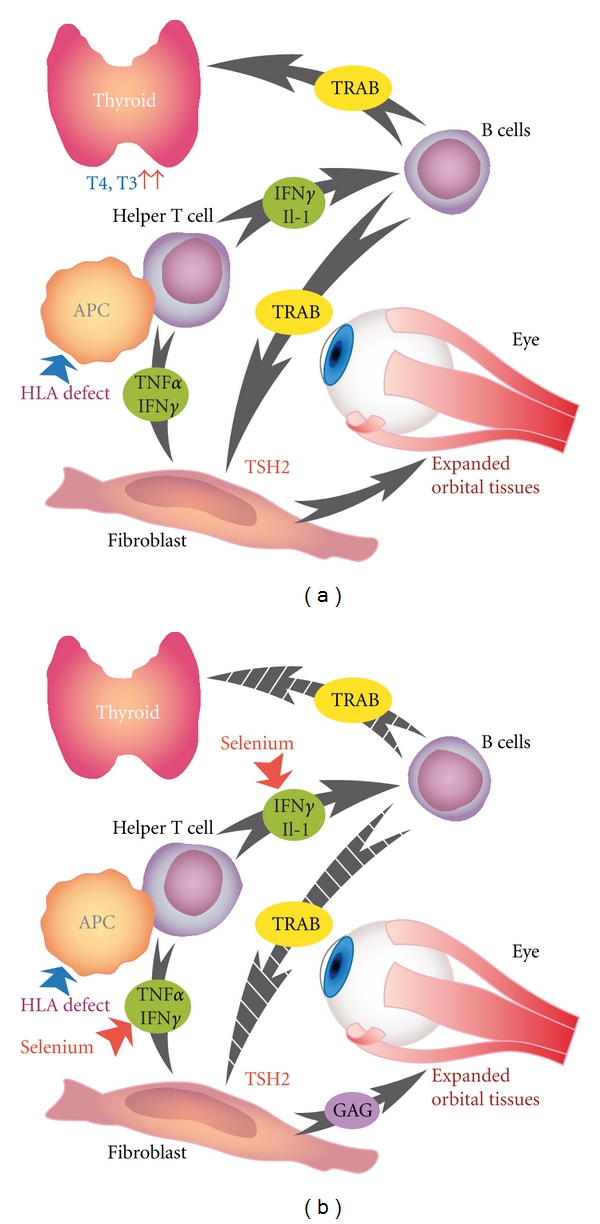
(a) Schematic presentation of the cascade of events in the pathogenesis of Graves' ophthalmopathy. Secretion of cytokines, such as IFN-*γ* and IL-2, by activated helper cells result in activation of B cells and secretion of TSH receptor antibodies. These bind to the TSH receptor in the orbital fibroblast and on the thyroid follicular cells, thereby, extending muscle enlargement resulting in oedema. (b) Selenium by suppressing cytokines production considerably attenuates the inflammation leading to alleviation of symptoms and signs. Abbreviations: HLA: human leukocyte antigen; APC: antigen presenting cell; IFN-*γ*: interferon-*γ*; IL-1: interleukin-1; TRAB: TSH-receptor antibodies; GAG: glycosaminoglycans.

Hyperthyroidism is caused by the binding of TSH-stimulating antibodies to the TSH receptor, a G-protein-coupled receptor. However, the first step in this process is considered to be precipitation by environmental factors of an HLA-related organ-specific defect in suppressor T-lymphocyte function [5]. This leads to decreased suppression of thyroid-directed helper T-lymphocytes which, in the presence of dendritic cells and macrophages, produce the cytokines *γ*-interferon (IFN*γ*) and interleukin-1 (IL-1), subsequently differentiating B cells to plasma cells and generating TRAB. Concomitantly, IFN*γ* enhances the expression of HLA-DR antigens on the surface of thyroid cells (Figure 1). Thus, IFN*γ* modulates the autoimmune process and, by stimulating chemokine production by thyroid follicular cells, contributes to the maintenance of the autoimmune process [10]. The contribution of dendritic cells and B cells is apparently crucial for the initiation of disease since they express the costimulatory molecules, CD80 and CD86, that are key triggers for the reaction of T lymphocytes to thyroid cell presenting antigens [4]. TRAB stimulate the TSHR on the thyroid follicular cells, resulting in increased thyroid hormone production, which may further reduce the number and function of suppressor T lymphocytes and stimulate helper T lymphocyte, thus, perpetuating the cyclicity of disease [4, 5].

GO is a complex autoimmune disease. Whereas the cycle of GD consists of two components, immunological and hormonal, that perpetuate the process, the progression of GD to GO, and rarely to dermopathy, is likely to be a positive feedback cycle composed of three interrelated components: mechanical, immunological, and cellular [5]. Comprehensive reviews on the pathophysiology of GO have recently been published [11–14]. Briefly, the loss of tolerance of T cells to the TSHR, via as yet unknown mechanisms, ignites the autoimmune process. The TSHR is internalized and presented by antigen-presenting cells to helper T cells. Subsequently, the TRAB, which are secreted by activated B cells, recognize the TSHR on the fibroblasts of the orbita, where they initiate the ocular changes [12, 13]. The fibroblasts have been recognized as target cells in GO. Orbital fibroblasts stimulated by IFN*γ*, tumor necrosis factor-*α* (TNF-*α*), growth factors and oxygen reactive species (ROS), secrete hyaluronic acid, and prostaglandin E_2_, known mediators of inflammation, while a subgroup may differentiate into mature adipocytes presenting TSHR [13, 14]. The subsequent proliferation of adipocytes and fibroblasts results in increased synthesis of glycosaminoglycans (GAG), which causes edema of orbital structures, extraocular muscle enlargement, and adipose tissue expansion; these events are constituting the signs of disease [15].

Concerning the recent enquiry as to whether autoimmunity against IGF-1R is primarily involved in the pathogenesis of GO, it is likely that it is not specific but instead constitutes a secondary reaction of the autoimmune process [16]

The mechanisms promoting oxidative stress have also been implicated in the pathogenesis of GO. Hyperthyroidism increases oxidants and decreases antioxidants leading to oxidative stress, this process is dominated by the production of ROS which have long been recognized as intermediates of various essential biological redox reactions [17, 18]. The adverse effects induced by ROS have been suggested as being partly responsible for the tissue injury. Mitochondria are a major source of superoxide anion (O_2_^−^) and hydrogen peroxides (H_2_O_2_), while a number of intracellular enzymes, xanthine oxidase being the best known, are involved in oxidation reactions in which molecular oxygen (O_2_) is reduced to O_2_^−^ [19].

Ongoing autoimmunity may contribute to increased oxidative stress even in euthyroid GD patients, while patients who have relapsed present increased markers of oxidative stress [20]. Moreover, the content of 8-hydroxy 2′-deoxyguanosine (8-OHdG), an important biomarker of oxidative DNA damage, was found significantly higher in orbital fibroblasts together with O_2_^−^ and H_2_O_2_, underscoring the major role that ROS play in the pathogenesis of GO [21].

Recently, increased 11*β*-hydroxysteroid dehydrogenase (11*β*-HSD1) expression, induced by cytokines, was described in orbital adipose cells, a condition leading to elevated local generation of cortisol by 11*β*-HSD1, which may suppress cytokine synthesis and resolve the inflammation [22]. 11*β*-HSD1 activates cortisone to cortisol in peripheral and visceral adipose tissues. According to the authors, since failure to produce adequate levels of local glucocorticoids in the orbita may signify persistence of the disease, 11*β*-HSD1 could provide a new therapeutic target of disease [22].

## 3. Presentation and Treatment Novelties of GD and GO

TRAB levels in serum are pathognomonic for GD, predicting the course of disease and response to antithyroid treatment; they do not, on the other hand, predict the development of GO [23]. In conjunction with the high levels of TRAB, the risk of relapse is related to young age, male gender, and large goiter [24]. Tobacco smoking has been consistently linked to development or deterioration of GO [8, 25]. Since RAI treatment for GD is associated with a worsening of GO, patients, and particularly those who are smokers, should be administered oral steroids [26]. Interestingly, a recent study from Varese has suggested that steroid prophylaxis can be achieved by applying lower prednisone doses, that is, 0.2 mg/kg BW, than had previously been reported [27]. Moreover, RAI when applied for treatment for GD results more frequently in aggravation or appearance of GO than after antithyroid treatment [28]. Nevertheless, choice of the best treatment for hyperthyroidism in patients with active GO remains a dilemma [29]. In a recent prospective analysis of the data of 108 patients with Graves' hyperthyroidism and severe orbitopathy, it was reported that prolonged treatment applying partial block therapy with low-dose thionamides plus LT4, over a median duration of 80 months, led to euthyroidism and stabilized the orbitopathy [30]. Within this context, a retrospective study proposes block-replacement treatment of GD patients with GO as a feasible treatment option until the orbitopathy becomes inactive, and no further treatment is required [31]. 

Neither antithyroid drug treatment nor thyroidectomy has any impact on the course of GO, and treatment in the active phase is based on the clinical activity score (CAS) [32, 33]; introduced by Mourits et al. in 1989, the CAS remains a reliable and easily applied scoring system enabling the classification of patients into those with active or inactive disease [33].

Recently, rituximab, a CD-20 antibody which blocks the differentiation of B cells and potentially inhibits B-cells-mediated immunity, was applied with encouraging results in patients with GO [34]. The compound was shown to improve GO without, however, affecting the TRAB levels [35]. Serum cytokine IL-6 levels did not change, while chemokine ligand 10 (CXCL10) increased at B-cell depletion.

Based on the knowledge of the crucial role of the oxidants in the pathogenesis of GD as well as in the development of GO, several studies have been conducted administrating antioxidants as the treatment modality in patients with GD and GO.

In a nonrandomized study, 82% of the 11 patients with active GO responded to antioxidant treatment with nicotinamide and allopurinol as compared to only 27% of the control group. Soft tissue inflammation parameters responded better than any other component of disease [36].

Supplementation with a mixture of antioxidants, including selenium, beta-carotene, and vitamins C and E, in addition to methimazole, in 29 patients with GD led to euthyroidism faster than in 28 patients taking only methimazole and who served as the control group [37]. Serum selenium levels as well as glutathione peroxidase activity were statistically significantly elevated in the supplemented patients, validating treatment with antioxidants, especially when this incorporated selenium.

In a more recent, randomized, double-blind, and placebo-controlled study recruiting 159 patients with mild GO, the effects of selenium administration for 6 months in the form of selenite were assessed versus an anti-inflammatory agent [10]. Selenium improved quality of life and significantly slowed the progression of GO, while it greatly decreased the CAS when compared with the pentoxifylline or placebo group. A 6-month followup confirmed the results of the 6-month treatment. The authors hypothesized a reversal of the disturbed antioxidant-oxidant balance in GD and GO although the exact mechanisms of selenium action are not elucidated.

In another study assessing the selenium levels in patients going into remission (*n* = 24) and relapses (*n* = 59), no statistically significant differences were detected between the two groups. However, patients in remission of GD had the highest (>120 *μ*g/L) serum selenium levels, while it is of interest that TRAB levels and selenium were negatively correlated [38].

## 4. Mechanisms of Selenium Action in GD and GO

Selenium is vital for a wide range of biological processes; hence, the state of “selenostasis” is essential for wellbeing and human health [39]. The many biological and clinical benefits conferred by selenium are achieved by virtue of its remarkable antioxidative effects mediated mainly by the selenoproteins GPx and TRx reductase. TRx is a stress- and iodine-induced protein, possessing strong redox activities, and it has been postulated that it may be implicated in the regulation of T3 production in GD. It has been reported highly produced in GD and expressed in the thyroid follicular cells. Nevertheless, its precise role, though of considerable interest due to its characteristics, remains as yet unraveled [40].

The hypermetabolic state in acute GD, the intracellular ATP, and increased oxygen consumption lead to mitochondria dysfunction, which generates ROS and disrupts the oxidant and antioxidant balance, thereby, causing oxidative stress and tissue injury [41]. By activating GPxs, selenium ignites the “second line” of antioxidant defense, behind the enzymatic “first line” defense system composed of the superoxide dismutase (SOD) and catalase (CAT) [42]. Thus, SOD and CAT synthesize an efficient antioxidative mechanism capable of neutralizing the biologic effects of free radicals; when this mechanism is saturated, the “second line,” regulated by selenium availability, is activated. Experimental studies in hyperthyroidism have documented an enhanced activity of the TRx and GPx systems, stimulated by the calcium phosphatidylinositol cascade which is usually activated in hyperthyroidism, as well as increased levels of SOD and of glutathione in erythrocytes [43, 44]. These findings provide evidence of an upregulation of the antioxidative and protective systems in acute GD, depending, however, on the duration and severity of the disease; these system(s) might become saturated, following which supplementation or nutritional intervention is required.

The induced oxidative stress enflames lipid peroxidation and activates various inflammatory pathways. ROS may stimulate the NF-*κ*B pathway, a cornerstone of immune and inflammatory response, which has been associated with increased production of TNF-*α* and IL-6 cytokines [45]. Selenium inhibits NF-*κ*B from binding to its gene promoters and consequently diminishes cytokine production and attenuates the inflammation; by contrast, selenium is likely not to interfere with the translocation of NF-*κ*B and its subunits to the nucleus [46]. This could be one of the most important anti-inflammatory effects of selenium supplementation and thus be of potential benefit for patients suffering from GD and, especially, GO.

In GO, the balance of T helper (Th) 1/Th2 lymphocytes shifts to a prevalence of Th1 type CD4+, which plays a pivotal role in the development of disease [47]. Consequently, the ratio Th1/Th2 has been proposed as a biomarker of disease activity and as a target for specific immune therapy of GO. The subsequent overproduction of cytokines, such as TNF-*α* and IFN*γ*, sustains the inflammatory process. It is of interest that treatment with a mixture containing selenium-suppressed Th1 while upregulating Th2 [48]. Th1 predominate in eye muscles (EM) and IFN-*γ*, TNF-*α*, IL-1*β*, and IL-6 mRNA have been abundantly detected in EM in contrast to orbit fat where IL-4 and IL-10 mRNA, with significant variations within patients, were more frequently detected [49]. Thus, mediated by the suppression of Th1-like cytokines, selenium alleviates the soft tissue inflammation and improves eye motility.

ROS, such as H_2_O_2_, may also activate p38 mitogen-activated protein kinase (p38MAPK) and induce expression of high levels of cyclooxygenase (COX)-2; this reaction is depending on the severity of GO, in orbital fibroadipose tissues [50]. Recently, it has been shown that selenium was able to reduce H_2_O_2_-mediated expression of COX-2 in vascular endothelial cells by inhibiting the p38 MAPK pathway [51].

In summary, selenium influences the inflammatory process in GD and GO by inhibiting various pathways though its mechanism of action is not completely clarified. It is nonetheless possible that, in synergy with antithyroid drugs or immune modulators, selenium might offer an alternative therapeutic approach in patients with severe disease. It also remains to be established whether enforced nutritional supplementation has the same effects and whether long-term selenium administration in the form of selenomethionine or as nutritional intervention may have an impact on the incidence of relapse of GD and GO.
